# Contribution to the reproductive ecology of *Notoscopelus resplendens* (Richardson, 1845) (Myctophidae) in the Central-Eastern Atlantic

**DOI:** 10.1038/s41598-020-72713-0

**Published:** 2020-09-25

**Authors:** A. N. Sarmiento-Lezcano, R. Triay-Portella, A. Guerra-Marrero, D. Jiménez-Alvarado, U. Rubio-Rodríguez, R. Núñez-González`, F. Bordes, J. J. Castro

**Affiliations:** 1 Instituto Universitario EcoAqua, Universidad de Las Palmas de Gran Canaria, Campus de Tafira, Canary Islands, 35017 Las Palmas de Gran Canaria, Spain; 2 Grupo Ecología Marina Aplicada y Pesquerías, Instituto Universitario de Investigación en Estudios Ambientales y Recursos Naturales I-UNAT, Universidad de Las Palmas de Gran Canaria, Las Palmas de Gran Canaria, Campus de Tafira, 35017 Las Palmas, Spain; 3 Instituto Politécnico Nacional, Departamento de Pesquerías y Biología Marina, CICIMAR-IPN, Av. Instituto Politécnico Nacional s/n, Col. Playa Palo de Santa Rita, 23096 La Paz, BCS Mexico

**Keywords:** Ecology, Animal migration, Ecosystem ecology, Developmental biology, Ichthyology

## Abstract

*Notoscopelus resplendens* is an abundant myctophid in the region of the Central-Eastern Atlantic. As with a majority of other myctophid species, this species performs vertical migration, playing a key role in the oceanic food web and in carbon sequestration. We examined the reproductive biology of *N. resplendens* based on 579 specimens caught between 1997 and 2002 off the Canary Islands. We found that the maximum standard length (*SL*) was lower than the size reported by other authors. The sex ratio was not different from 1:1. The average size at first maturity (L_50_) was higher in females (60.34 mm SL) than in males (56.61 mm SL). The gonadosomatic index (GSI) at 50% sexual maturity in females was higher than that in males. The reproductive activity was observed from January to April, while from May onwards, the majority of fish caught were in the process of maturation. The macroscopic scale of maturation was validated through the histological analysis of the ovarian development. The batch fecundity was related to the standard length, with an average of 1068.69 ± 369.84 eggs/spawn. These first data obtained for *N. resplendens* indicated that it is a batch spawner with asynchronous ovarian development.

## Introduction

The mesopelagic domain (200–1000 m depth) has massive concentrations of organisms and is thus considered one of the areas with the highest biomass in the ocean. Some of those organisms are aggregated in the mesopelagic layer called the Deep Scattering Layer (DSL), which is an area of bioacoustic dispersion formed mainly by crustaceans, cephalopods and fishes^1,2^. Approximately 40% of the organisms in the DSL feed between twilight and dawn in the epipelagic zone (0–200 m depth)^3,4^, playing a key role in the oceanic food web^5–7^ and in carbon sequestration^8,9^.

These mesopelagic fishes are the dominant species in the ocean and are the vertebrates with the largest biomass on the planet^10^. The total biomass of these species has been estimated at approximately 1000 million tons^11^, but these values may be underestimated by one order of magnitude^12^. Moreover, the estimated contribution of these species to deep water respiration would be approximately 10%^12^, and thus, their role in ocean ecosystems and their contribution to ocean biogeochemical cycles have vital importance.

Myctophidae, commonly called lanternfishes, is the main family of mesopelagic fish and is present in all the world's oceans^13^. Usually, the myctophids are distributed throughout the water column, but they are more frequently found between 200 and 1000 m in depth (the mesopelagic zone) as part of the DSL^14^. They comprise approximately 35 species in 12 genera, and their estimated biomass may substantially exceed 70–200 million tonnes (Mt)^12,13,15^. In addition to their high biomass and mobility, several authors refer to myctophids as the largest species in the mesopelagic zone (generally between 2 and 15 cm in total length)^12,16^.

Myctophids play an important role in energy transfer in pelagic ecosystems, linking the planktonic organisms such as copepods, ostracods and larvaceans^17,18^, with pelagic fish^19^, cephalopods^20^, seabirds^21^ and marine mammals^22^. Despite the intense predation they support, lanternfishes are highly abundant^23^, and it is important to understand their population dynamics, particularly their reproductive biology^12^. Moreover, as a potential fishery resource^24^, interest in the biology, ecology and population dynamics of these mesopelagic fishes is progressively increasing^25,26^.

Studies of the reproductive biology of species of the Myctophidae family are limited^27–30^ and frequently have not considered fecundity type (determinate or indeterminate), which provides important information^31–35^. Typically, studies of the reproductive biology of the myctophids include information about sex ratio, oocyte development, sizes at sexual maturity, spawning seasons, fecundity, and spawning strategies^23,28,30,34–40^, but this information is only available for a very few species.

*Notoscopelus resplendens* (Richardson, 1845) is a circumglobal species in tropical through temperate seas^41^ that forms a part of the DSL community. In the Atlantic, it is distributed from southern Britain to the Southern Ocean and from Newfoundland to Rio de la Plata^14^. Like many other mesopelagic species^42^, *N. resplendens* migrates from the depths to the surface at night, crossing water with very different features^43^. This species exhibits its highest abundance levels in the Eastern Atlantic region along the African coast, including the Canary Islands^44–46^, in ecoregion 24, as described^47^ in a global biogeographic classification of the mesopelagic zone. Some authors^6^ have indicated that *N. resplendens* in the Kuroshio–Oyashio transition zone is one of the dominant components of the mesopelagic fish. However, no information is available on the reproductive biology of this species. Age-based life-history parameters have been estimated for this species^46^, contributing age at first maturity and spawning period data. The spawning season seems to be from December to March, based on back calculating the hatching date from the daily growth increments, and the age at first maturity was 1.7 years for males and 2.05 years for females^46^.

In spite of the abundance and importance of *N. resplendens* in the mesopelagic ecosystem, its biology and ecology are poorly known, and most of the available information is related to its growth, reproduction and life cycle in the Pacific Ocean^48^. In this sense, it is an oceanic and mesopelagic species that performs diel migrations from as deep as 650–1000 m during the day up to the surface through 300 m during the night for feeding purposes, although the larvae and transforming individuals are non-migratory^45^. Therefore, the aim of this study is to provide additional information on the reproductive biology of this species in the Central-Eastern Atlantic, near the Canary Islands, particularly that related to oocyte development (histological analysis) and reproductive parameters [i.e., length frequency distributions, condition factor (K), sex-ratio, length at maturation, spawning season, and batch fecundity] to estimate the reproductive strategy. These data are important for understanding the population dynamics where other biological and fishery estimates are unavailable for this species.

## Material and methods

**Figure 1:**
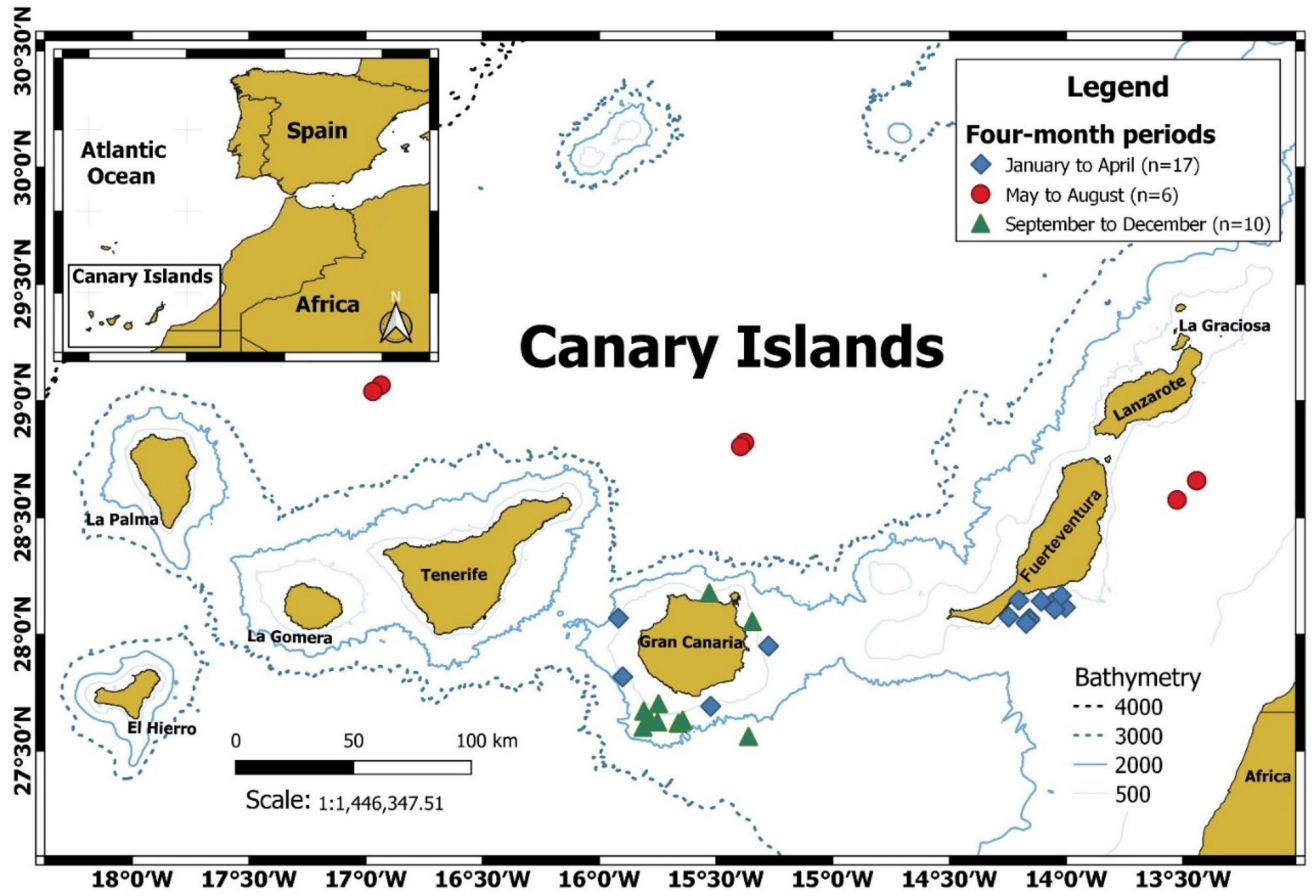
Sampling area conducted by the B/E La Bocaina between 1999 and 2002. Map created in QGIS Development Team (V.3.12.1 https://www.qgis.org/es/site/)^62^.

The study was based on the analysis of 579 specimens of *N. resplendens* caught during 4 cruises of the B/E “La Bocaina” between 1997 and 2002 (because the cruises did not cover all months of all years and to facilitate data analysis, the samples were grouped by 4-month periods, assuming no significant variation among years), off the Canary Islands (Central-Eastern Atlantic) (Fig. 1). The fishes were caught with a commercial semi-pelagic trawl net with a cod-end with 5 mm mesh size, but on the last cruise (2002), this mesh size was increased to 10.4 mm^49^. The hauls were conducted horizontally during the diurnal and nocturnal periods at a depth range between 13 and 1577 m. Fishing operations were monitored using acoustic telemetry, with a net-sounder SCANMAR, which provided information on the depth and the vertical and horizontal opening of the trawl mouth. Characteristics of the vessel and the net and a description of the fishing operations are given in other published works^50,51^.

Captured fishes were identified to the lowest possible taxon and stored in 70% ethanol for later analysis. As proposed in a previous study^44^, the number of gill rakers was used to identify individuals of the *Notoscopelus* genus. Once in the laboratory, for each fish, the total length (*TL*, 0.01 mm) and standard length (*SL*, 0.01 mm) were recorded using a digital calliper, and the whole body weight (*BW*, 0.01 g) and gonad weight (*GW*, 0.0001 g) using a digital balance. Generally, this species is captured using a midwater trawl net with a reduced mesh size, causing the loss or breakage of parts of the organisms' bodies (such as the caudal fin). For this reason, a consensus has been reached to use the standard length measurement (SL, from the tip of its nose to end of its last vertebrae, i.e., excepting the caudal fin). Here, the SL–TL relationship was estimated for all organisms using a linear equation to transform the published data of other authors (expressed in TL) and can be compared with our results. Individuals were then dissected, and the digestive tract, stomach and gonads were removed.

The specimens were grouped into length classes of 10 mm increments, and the length frequency distribution was analysed as percentages by 4-month periods [January to April (n = 191), May to August (n = 233) and September to December (n = 155)].

The relative fatness (energy reserves) and its relation to reproduction were estimated using the condition factor (*K*)^52^, which, as a measure of the physiological changes that occur in the organisms, was calculated for each individual as K = (BW/SL^3^) × 100.

Sex was estimated from 518 samples due to external body dimorphism. Males present a large supra-caudal gland that allows them to be differentiated externally from females. The sex was confirmed after dissecting the fish and observing the gonads macroscopically. However, the state of maturity was determined for only 459 individuals through gonad macroscopic observation, following the classification criteria for fishes^53^ to classify these stages as immature, developing, spawning capable, regressing and regenerating. The macroscopic scale of maturity was validated with histological analysis, including all maturity stages, considering the standard terminology for describing reproductive development in fishes^53^. A random stratified sampling strategy was applied. For each macroscopic ovary stage, 10 gonads each were selected and processed histologically for each ovary maturity development stage.

The sex-ratio was calculated for the whole sampling period and for the three 4-month periods (quarters) considered, and whether these rates were significantly different from the theoretical ratio of 1:1 was estimated using a chi-square test (χ^2^-test). For mature individuals (n = 187), the maturity ogives, lengths at first (*L*_50_) and massive maturity (*L*_95_) for both sexes, and the percentage accumulated by length class of mature individuals were calculated. The data obtained were fitted to a normal cumulative curve by iterative nonlinear regression. Furthermore, a comparison of means test was performed to detect possible differences between the *L*_50_ of males and females. The data were fitted to a sigmoid function as follows:

$$ P_{r} = \frac{100}{{1 + e^{{ - r \left( {L - L50} \right)}} }}, $$

where *Pr* is the percentage of sexually mature individuals, *r* is a constant that indicates the slope of the curve, *L*_*50*_ is the length at which 50% of individuals were mature, and *L* is the fish length for which the *Pr* is calculated.

To determine the spawning season, the gonads of individuals were monitored over time, and the average values of the Gonadosomatic Index (*GSI*) were calculated for each specimen, as the relationship between *GW* and *BW–GW*^54^:

$$ GSI = \left( {\frac{GW}{{BW - GW}}} \right)100. $$

Another method used to determine the spawning season is based on the temporal variation in the frequency of individuals in each stage of maturity, which was determined by macroscopic observation of the gonads. To do this, the number of fish in each stage of maturity and their frequencies were calculated. To estimate GSI at 50% maturity (GSI_50_), a logistic function was fitted to the fraction of mature fish per 0.5 GSI interval, for males and females, using a nonlinear least-squares regression. The logistic equation was:

$$ PM_{GSI} = \frac{100}{{1 + e^{{ - a \left( {GSI - b} \right)}} }}, $$

where *PM*_*GS*I_ is the percentage mature at GSI, a is the slope, and b is the *GSI*_50_. The minimum size at maturity of males and females was taken as the smallest specimen with a GSI over *GSI*_50_ based on the GSI–SL relationships^40^.

A total of 50 ovaries were fixed and preserved in 4% buffered formaldehyde for histological analysis to verify the previously assigned macroscopic maturity stages. For this, the fixed tissues were dehydrated in a series of ethanol solutions, cleared in isoparaffin H, and then embedded in paraffin in a vacuum chamber. Slices of tissue were sectioned at 4 µm and stained with Harris haematoxylin followed by eosin counterstaining^55^. To describe the scale of gonadal maturity, the standardized nomenclature^53^ was used. The size of the oocytes within the four ovarian developmental stages were determined to describe the type (synchronous or asynchronous) of maturation and spawning based on ovaries that were previously processed histologically. The oocytes were removed from the ovary then placed in a vial and pipetted vigorously or full separation. The contents of the vial were then poured into a counting chamber, and the isolated oocytes were measured. The first 100 oocyte diameters were measured to the nearest 0.01 mm and classified based on histological correspondence images.

Finally, 84 samples of female gonads were collected to estimate the batch fecundity (BF)^56,57^ through the gravimetric method, which was calculated for each female as the number of oocytes per unit weight multiplied by the total ovarian weight^40^.The proportion of the subsample from which the oocytes were extracted was evaluated with a target coefficient of variation (CV) of oocytes per unit weight of less than 5%^58^. The oocytes were manually released from the ovarian stroma and then counted using a stereoscopic microscope. Ovaries that did not contain early stage postovulatory follicle complex (POFs) were used because the presence of these indicate that some eggs have been already released^59^. In these ovaries, the oocytes at the most advanced stages, primarily Vtg3 and hydrated oocytes (*H*), were counted to estimate the batch fecundity^56,57^.

The statistical analysis was performed using the R programming language (V.3.6.0)^60^. Batch fecundity data from other studies are also shown for comparison purpose^40,61^. We obtained the values from the data points of figures using GetData Graph Digitizer V.2.26. The sampling map was generated using the geographic information system QGIS Development Team (V.3.12.1)^62^.

### Ethical approval

The sampling was approved by the "Viceconsejería de pesca del Gobierno de Canarias" and the samples were obtained through commercial fishing trawls. We worked with preserved fishes in the laboratory.

### Sampling and field studies

All necessary permits for sampling and observational field studies have been obtained by the authors from the competent authorities and are mentioned in the acknowledgements, if applicable. The study is compliant with CBD and Nagoya protocols.

## Results

### Length frequency distributions and sex ratio

**Figure 2:**
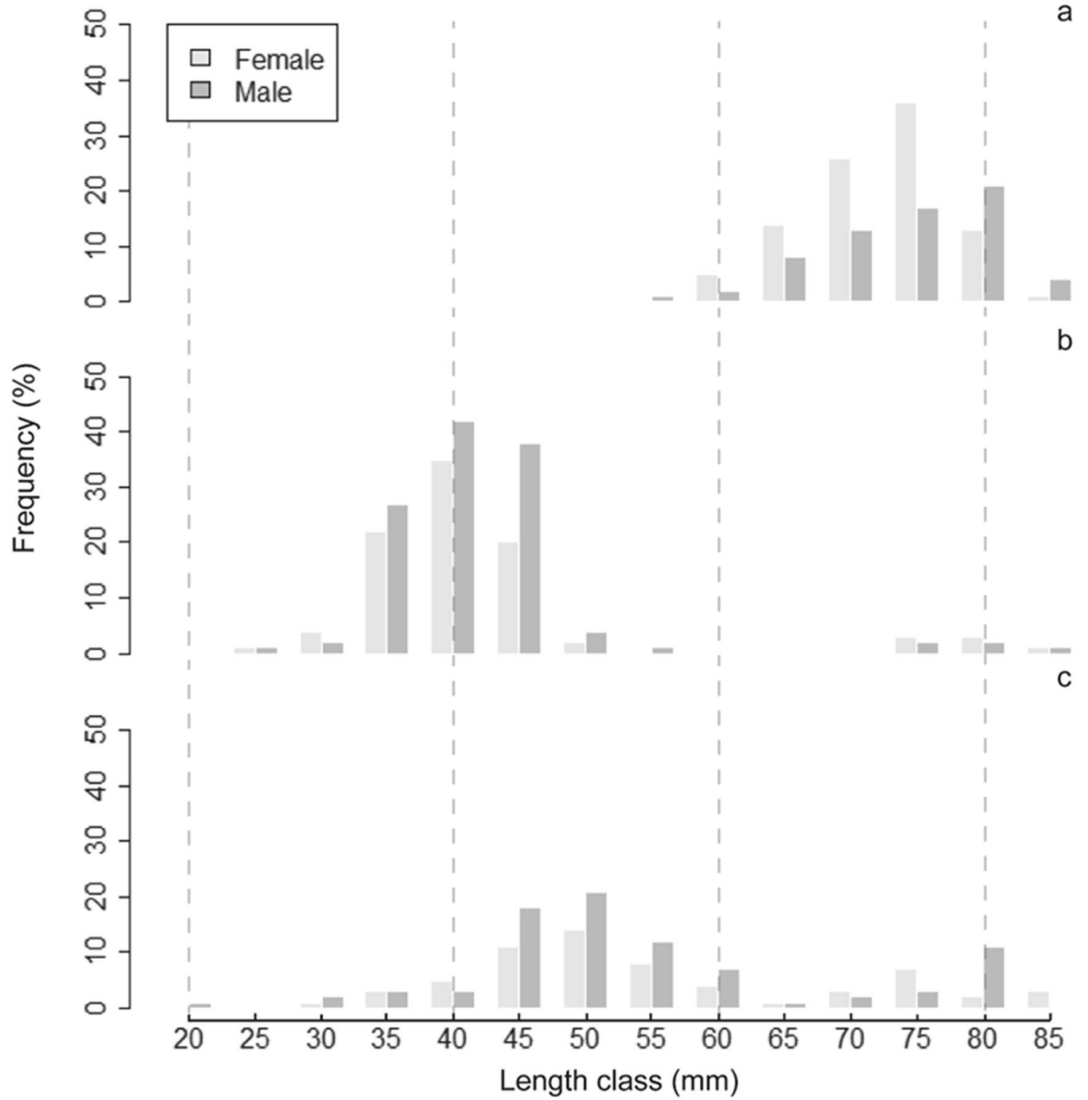
Distribution of size frequencies by sex: (**a**) January to April, (**b**) May to August, and (**c**) September to December.

The length frequency distribution showed the presence of two groups of lengths during the sampling period. From May to December, individuals of relatively small size (smaller than 60 mm SL) predominated, while larger fish (larger than 65 mm SL) were more abundant from January to April (Fig. 2).

Considering the entire sampling period, the sex ratio of *N. resplendens* was not different than 1:1 (1:0.92 χ^2^-test, χ_0_^2^ = 0.93; N = 518; p > 0.05). From January to April, females significantly predominated in the size class between 60 and 75 mm SL (1:1.43, χ_0_^2^ = 5.22; N = 161; p < 0.05; Fig. 2a). From May to August, males predominated in the length range between 35 and 45 mm SL (1:0.76, χ_0_^2^ = 3.98; N = 211; p < 0.05; Fig. 2b). From September to December, the sex ratio was not different from 1:1 (1:0.74, χ_0_^2^ = 3.31; N = 146; p > 0.05; Fig. 2c). The total length (TL) and standard length (SL) were highly correlated (SL = 1.01 + 0.89·TL; r^2^ = 0.99; p < 0.001).

### Length at maturation

**Figure 3:**
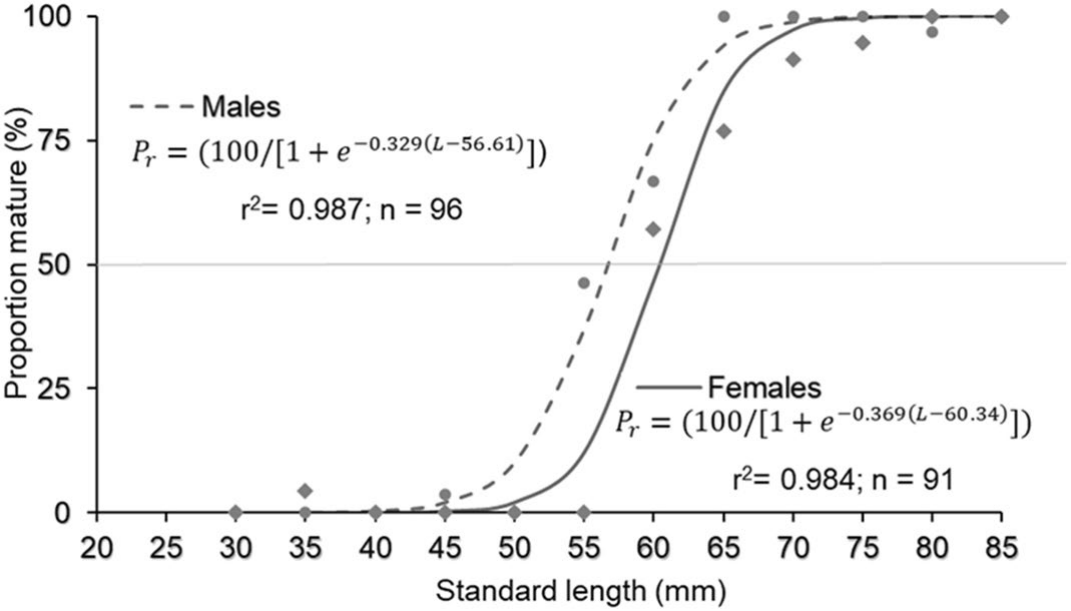
Maturity ogive for males and females of *N. resplendens*.

Among all fish sexed (n = 518), 36.10% of them were mature. Although the caudal gland in males was observed beginning at 25.29 mm SL, the L_50_ was estimated to be 56.61 mm SL (n = 96). However, females (n = 91) reached the L_50_ at a greater length (60.34 mm SL) (ANOVA, F = 4.829; p-value < 0.05). L_95_ values were estimated to be 65.12 mm and 68.83 mm SL for males and females, respectively (Fig. 3).

### Condition factor (K) and spawning season

**Figure 4:**
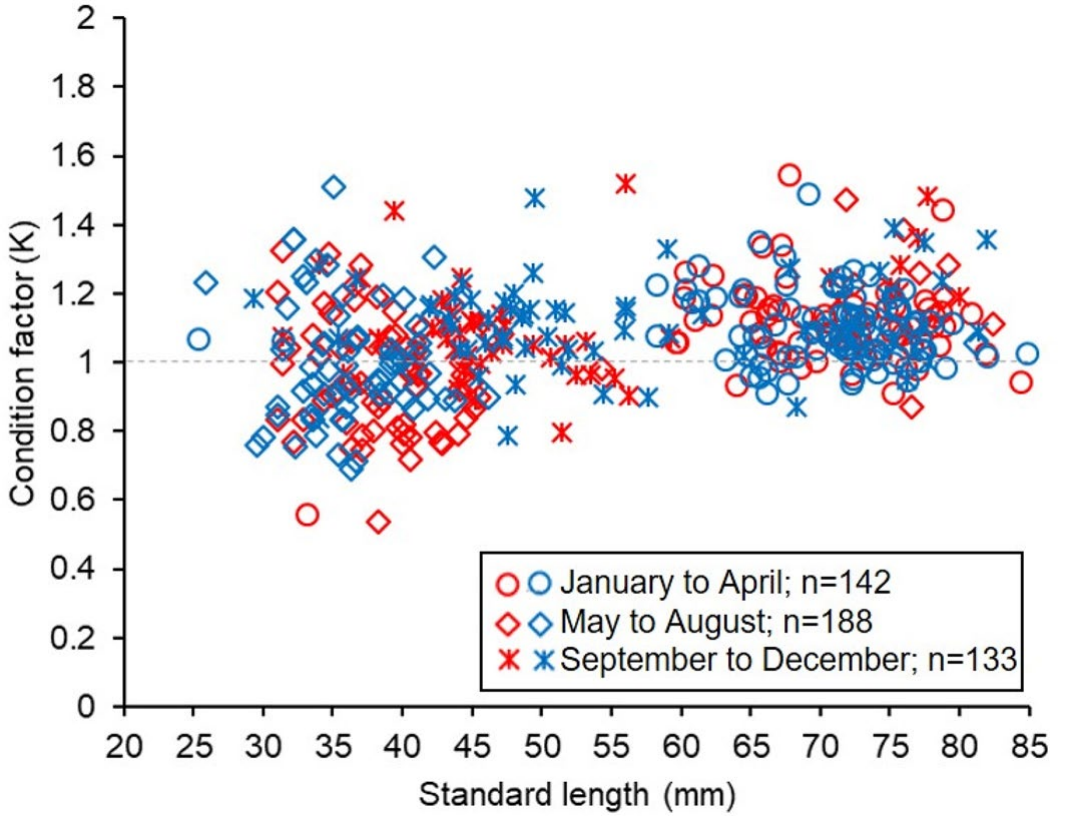
Variation of relative condition factor (K) for each four-months period throughout the available range of lengths available for *N. resplendens.*(Red dots: Females individuals; Blue dots: males individuals).

K values ranged between 0.539 and 1.627 (Fig. 4) in the length range between 25 and 85 mm SL. Most fish captured from January to April had K values greater than 1, particularly in the length group larger than 60 mm SL. However, from May to December, these relatively high values of K were observed particularly in fish smaller than 60 mm SL. An ANOVA comparing the differences in K among sampling periods showed significant differences (F_0.05,2,>200_, = 17.12, p < 0.001), and the Post-Hoc Tukey test showed differences between January to April–May to August (p < 0.001) and May to August–September to December (p < 0.001). However, there were not significant differences between males and females in all sampling periods (F_0.05,2,>200_, = 0.218, p = 0.641) and for each period (January to April: F_0.05,2,>200_, = 0.141, p = 0.708; May to August: F_0.05,2,>200_, = 0.900, p = 0.344; September to December: F_0.05,2,>200_, = 1.298, p = 0.257).

**Figure 5:**
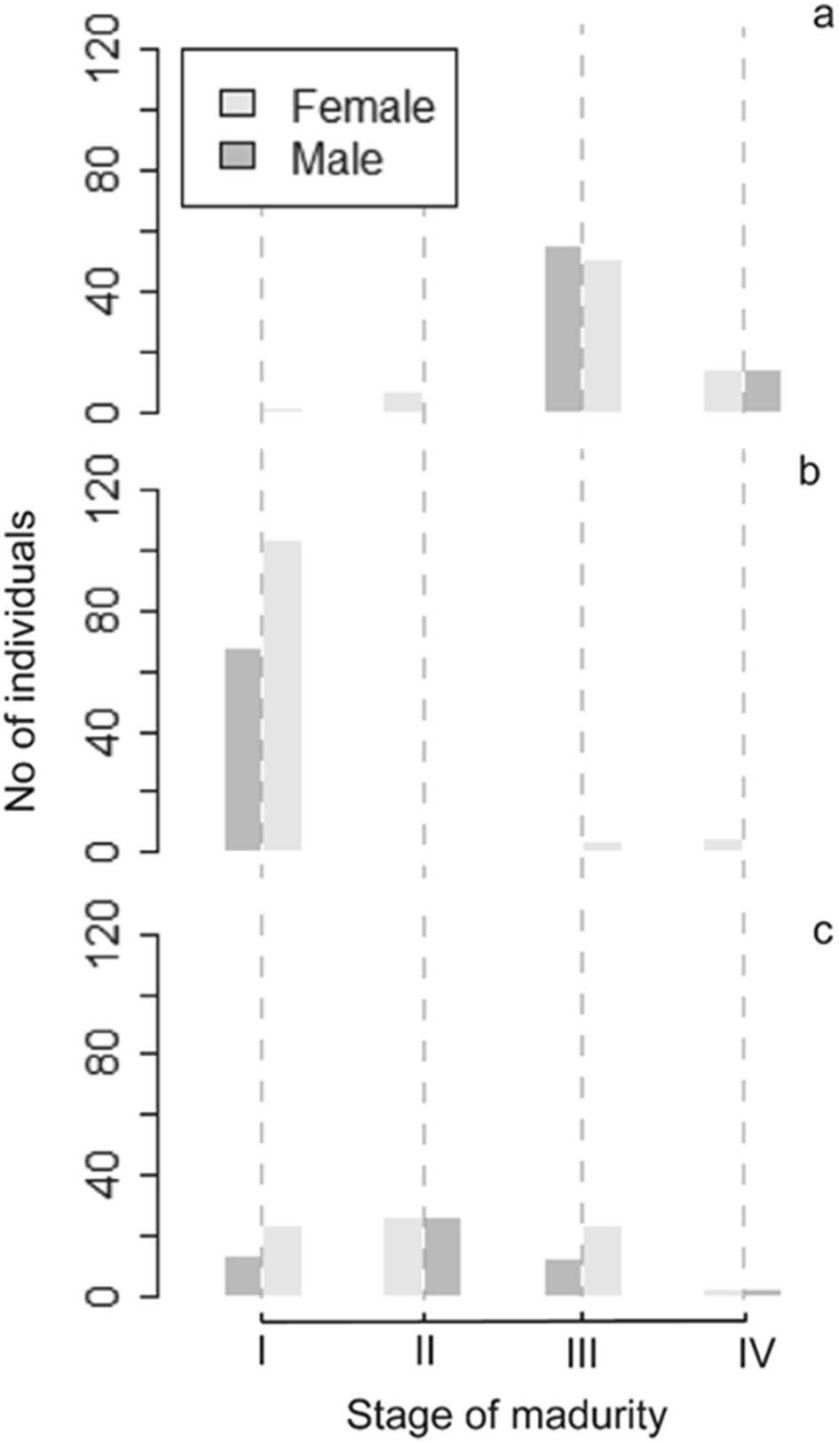
Variation state of maturity for males and females: (**a**) January to April, (**b**) May to August, and (**c**) September to December.

Fish at stages III (Spawning) and IV (Regressing) (Fig. 5) were recorded during the whole sampling period, but from January to April (n = 134) and from September to December (n = 43), a relatively high proportion of spawning individuals were recorded (spawning takes place in winter and spring).

**Figure 6:**
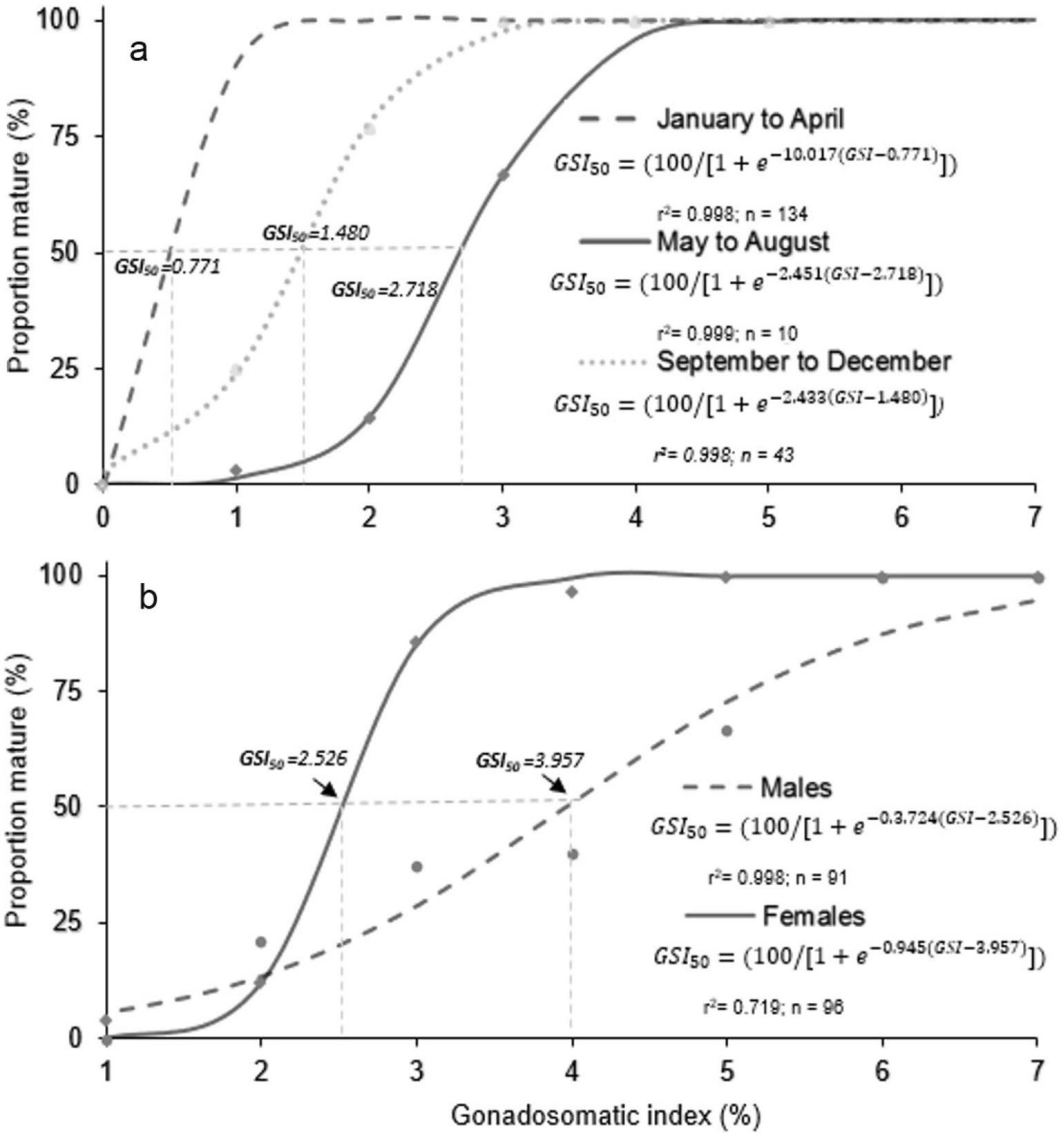
Relationship between gonadosomatic index (GSI) and percentage of mature individuals, (**a**) by 4-month period, and (**b**) by sex of *N. resplendens.*

**Figure 7:**
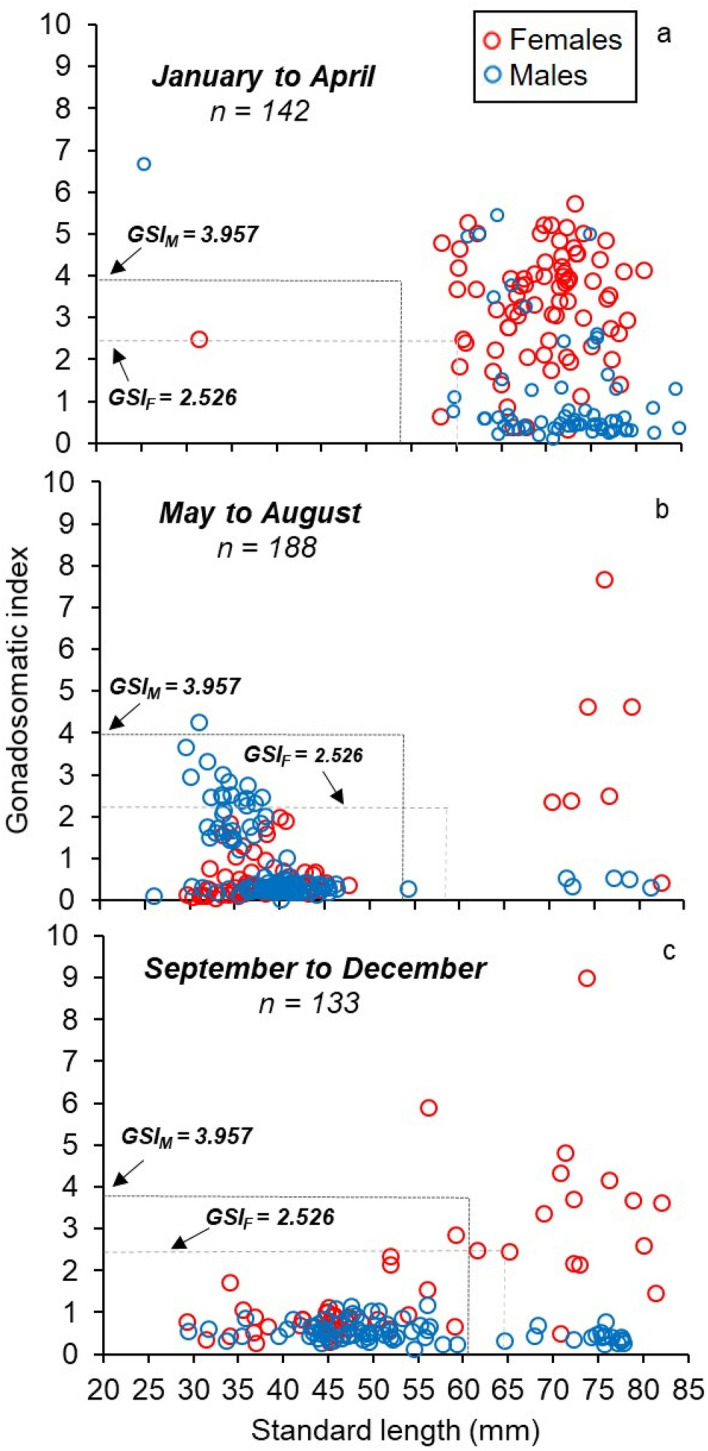
Relationship between standard length (SL, mm) and gonadosomatic index (GSI) of *N. resplendens* by each four-month periods. Horizontal dashed lines indicate the GSI at 50% sexual maturity (GSI50), and arrowheads on the horizontal axis indicate the minimum size at sexual maturity for each sex.

On the basis of the logistic function (Fig. 6a), the GSI_50_ values were 0.771 from January to April, 2.718 from May to August, and 1.480 from September to December (Fig. 6b). For males and females, GSI_50_ was 3.957 and 2.526, respectively*.* There were males larger than 50.61 mm SL and females larger than 60.34 mm SL with GSI values higher than GSI_50_ in all samples (Fig. 7). There were significant differences in GSI between males and females for January to April (F_0.05,1,140_, = 82.94, p < 0.001; Fig. 7a) and September to December (F_0.05,1,131_, = 33.78, p < 0.001; Fig. 7c).

### Histological analysis

**Figure 8:**
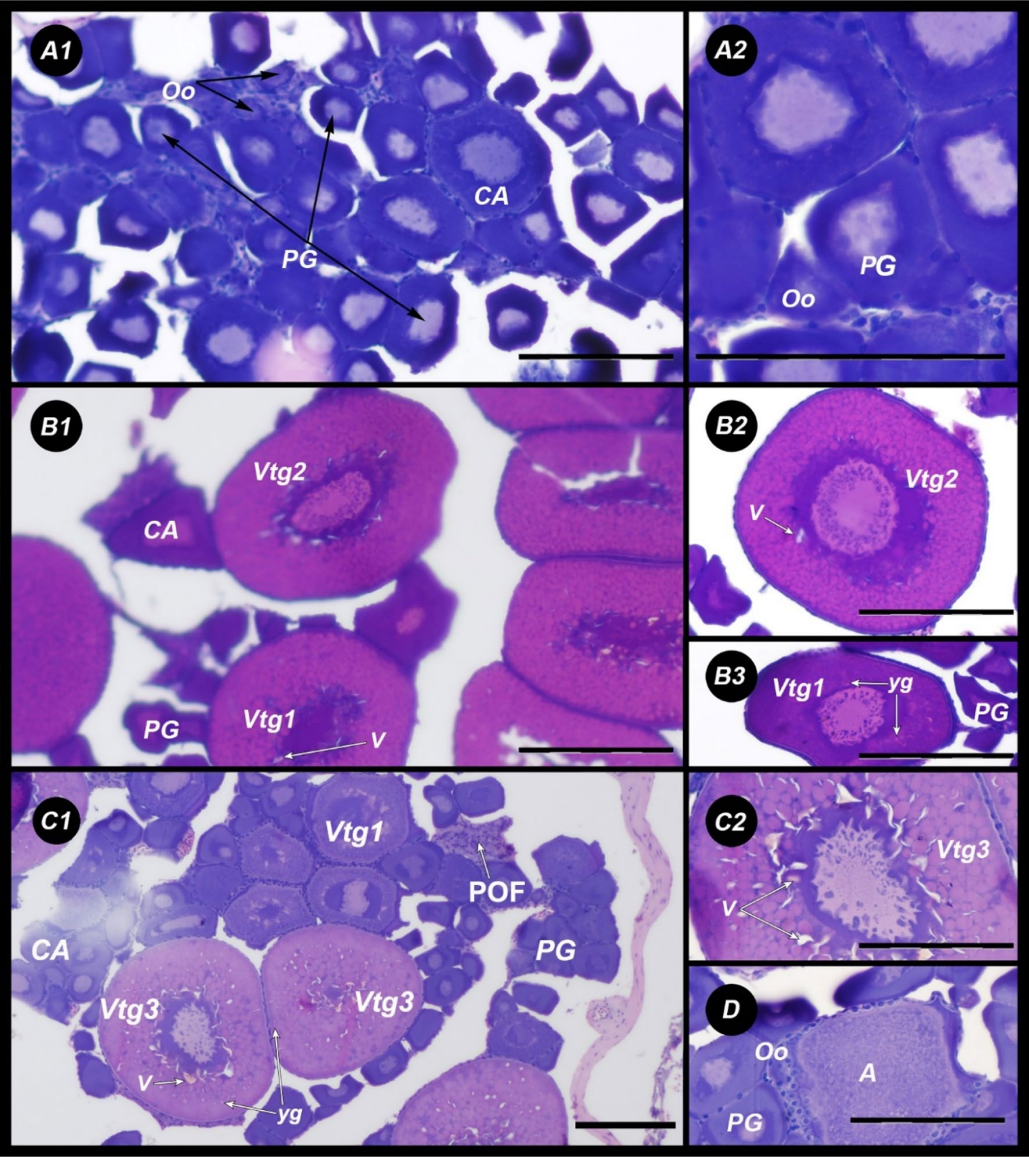
Histological sections of female gonads: (**A**) Immature: A1, A2 = Oo: Oogonia (< 15 μm), CA: cortical alveolar oocytes (35–70 μm), PG: Primary growth oocyte (20–35 μm); (**B**) Development: B1, B2, B3 = CA, PG, Vtg1 = primary vitellogenic (75–100 μm), Vtg2 = secondary vitellogenic (100–220 μm); (**C**) Spawning: C1, C2, C3 = Vtg1, PG, Vtg3 = tertiary vitellogenic (220–350 μm); and (**D**) Regression: Oo, PG, A = Atresia [Scale bar = 50 micron (μm)]. *V* yolk vacuoles, *yg* yolk granules.

The description of four different stages of development in the female gonads was performed, and the macroscopic observations were validated with gonad tissue histology. The standardize nomenclature^53^ was used to describe the ovarian development of *N. resplendens* in four reproductive phases described below (Fig. 8):

1. Immature phase—small ovaries, often clear, blood vessels indistinct. Only oogonia and PG oocytes present (Fig. 8A1–A2).
2. Developing phase—enlarging ovaries, blood vessels becoming more distinct. PG, CA, and Vtg1 oocytes present (Fig. 8B1–B3).
3. Spawning capable phase—large ovaries, blood vessels prominent. Individual oocytes visible macroscopically. Vtg3 oocytes present or POFs present in batch spawners (Fig. 8C1–C2).
4. Regressing phase—flaccid ovaries, blood vessels prominent. Atresia (any stage) and POFs present. Some CA and/or vitellogenic (Vtg1, Vtg2) oocytes were also present (Fig. 8D).

The analysis of the inner structure of oocytes in N. resplendence showed that Vtg1 oocytes were characterized by the presence of small vacuoles, the phases Vtg2 and Vtg3 were defined by the presence of vacuoles in the central and perinuclear areas of the oocyte, and of small yolk granules at its margin (Fig. 8).

Presence of oocytes in different videogenic state in the spawning capable phase determined that *N. resplendens* is a batch spawner with asynchronous ovarian development.

### Fecundity

**Figure 9:**
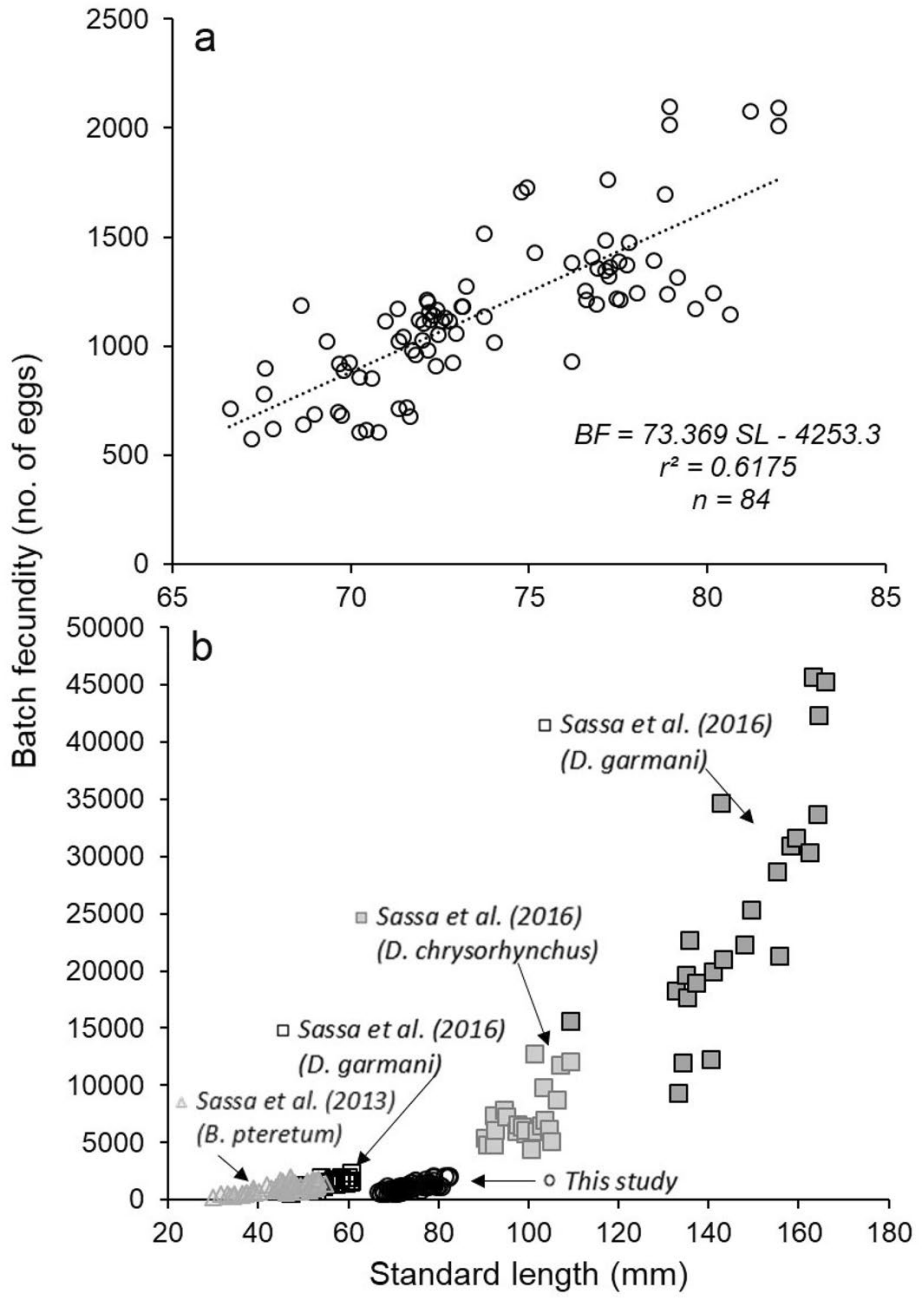
Relationship between the batch fecundity with the standard length for (**a**) *N. resplendens* and (**b**) other studies.

Batch fecundity (*BF*) was significantly related to standard length in mature females (n = 84), increasing linearly for standard lengths between 66.6 mm and 82.39 mm SL (ANOVA, F_2,83_ = 143.4 p < 0.01), with an average of 1068.69 ± 369.84 eggs by spawn pulse (range = 1089.24–1248.05) (Fig. 9a). As expected, the slope of the regression line of batch fecundity and SL of *N. resplendens* mature females was similar to those of *Diaphus garmani* and *Diaphus pteretum,* although the standard length of the latter two species was less than 60 mm SL (Fig. 9b). Statistically, the slopes for other species of *Diaphus* gender were significantly steeper than that of *N. resplendens* (ANOVA, F_4. 218_, F = 264.1; p < 0.01).

## Discussion

Information about the distribution and some age and growth parameters of *Notoscopelus resplendens* have been studied in the Central-Eastern Atlantic^14,46,49,63,64^; however, this is the first time that data have been reported for lengths at first (*L*_50_) and full (*L*_95_) maturity, spawning season and fecundity of this lanternfish species in this region (Table 1).

**Table 1:** Summary of the results obtained in this study for *N. resplendens* by 4-month period.

	Nº stations	Sex-ratio	LM (mm SL)	Inmature/mature ind. (%)	GSI_50_ (%)	N
January to April	17	1:1.43 (χ_0_^2^ = 5.22)	L_50_ (n = 187) 56.61^M^ 60.34^F^ L_95_ (n = 187) 65.12^M^ 68.83^F^	9.21/90.78^F^ 1.51/98.48^M^	0.771^G^ 2.526^F^ 3.957^M^	191^G^ 95^F^ 66^M^
May to August	6	1:0.76 (χ_0_^2^ = 3.98)		92.00/8.00^F^ 96.33/3.67^M^	2.718^G^ 2.526^F^ 3.957^M^	233^G^ 91^F^ 120^M^
September to December	10	1:0.74 (χ_0_^2^ = 3.31)		71.42/28.57^F^ 64.93/35.06^M^	1.480^G^ 2.526^F^ 3.957^M^	155^G^ 62^F^ 84^M^

*LM* Length at maturation, *N* number of individuals, Superscript (*F* Female, *M* Male and *G* Global).

*Notoscopelus resplendens* caught in Canarian waters ranged from 19.24 to 84.77 mm SL (0.050–5.660 g in weight), slightly larger than reported for individuals of its congeneric species *N. elongatus kroeyeri* in the Northeast Atlantic (18.81–67.76 mm SL, TL values converted with the SL–TL ratio estimated in this study)^31^. Although the length range of individuals depends on the fishing gear used, in this study, the length range of *N. resplendens* is within the limits previously described for mesopelagic fishes (18.81 and 135 mm of SL)^12,16^.

The proportion of males and females in the entire sample was a 1:1 ratio, but we did not find any significant differences in their proportionality according to season. In this regard, the greater number of females observed in January to April could help maximize the egg-producing biomass^34,65^, and the opposite tendency recorded during May to August, when males predominated, could enhance the likelihood of mating but at the expense of a decreased number of egg producers in the population^66^. These differences in the sex ratio during the year (or years) and even with depth intervals have been observed in several species of myctophids, such as *Benthosema pterotum*^67^.

The ratio of males to females decreased with SL, and most individuals in the largest class were females, as observed in many other myctophids^37,39,40^. This variation in the sex ratio with length might be related to faster growth and/or a longer life span of females in relation to males^65^. This has been documented in several myctophid species belonging to the *Notoscopelus* genus, where females grow faster than males and reach a larger maximum size^46,68,69^.

Previous studies have found that males *N. resplendens* begin to develop the large supra-caudal gland at 37.5 mm SL (41 mm TL), reaching sexual maturity at 59.65 mm SL (66 mm TL)^70^. Our data indicate that in the Canary Islands region this gland begins to develop at a smaller length than that reported by Hulley^13^ (25.29 mm SL, i.e., 27.14 mm TL), and consequently, the average length of first maturity was also lower (56.61 mm SL, i.e., 61.60 mm TL) than previously estimated by the abovementioned author. Sexual dimorphism in luminous organs is known in many myctophids^71,72^. These caudal organs are considered to be related to sexual recognition in myctophids because they can produce volleys of very fast and high intensity flashes^72,73^. Studies on *B. pterotum*^61^ suggest that this bioluminescent sexual signalling might be used to facilitate communication between sexes at night, and this is possibly related to more efficient mating.

In contrast, females of *N. resplendens* were mature at a larger size than males, with a length at first maturity of 60.34 mm SL. In other myctophid species, males mature at smaller sizes than females^29,30,34,65^. This would contribute to the greater biomass of mature females than males and to maximizing the egg-producing biomass.

In other studies of myctophid species, for example, in the *Diaphus* and *Benthosema* genera, the range of size at maturation is between 24.5 and 120 mm SL^38,40,61^, depending on the growth rate of the species and the region in which it is found. This could be the reason we found variability in the size of first maturity in the same species.

Seasonality in the spawning of many myctophid species has been reported in different areas of the world’s oceans, although year-round spawning has also been observed^30,61,74^. In Bermuda^75^, *N. resplendens* spawns from winter to spring, with a peak of intensity in winter and early spring. The abundance of post-larvae suggested that spawning began prior to January and reached a peak in late February–early March. In a similar way, most of the individuals of *N. resplendens* caught off the Canary Islands showed relatively high values of K and GSI from January to April (corresponding mainly to winter and early spring), predominantly in those larger than 60 mm SL, but at the end of spring and summer (from May to August), the proportion of individuals with K lower than 1 was significantly greater, particularly among smaller-sized fish. In this regard, the K variations in *N. elongatus kroeyeri* were related to the spawning season^31^. Additionally, for the latter species, found in the Mediterranean^76^, the K values declined during the spawning season, which occurs from winter to spring, with a peak in April.

Therefore, the lower K values observed in specimens of *N. resplendens* caught in March and May are related to the end of the reproductive season in the waters of the Canary Islands, during a similar period to that observed in the neighbouring waters of Mauritania, where spawning takes place in winter and spring^77^. Moreover, *N. resplendens* showed reproductive activity during winter and early spring (January to April), when the percentage of mature individuals was 70.2%, with reproductive activity occurring particularly in the larger length range.

In contrast, the histological analysis of the female gonads of *N. resplendens* showed oocytes in different developmental states, indicating that this species has asynchronous ovarian development with successive batch spawner seasons, as observed in other myctophids^28,38,39^. In addition, this species is characterized by intermittent spawning with long intervals between batches^77^. In the Canary Islands, batch fecundities vary from 578 to 2,122 eggs and progressively increase with fish length, from 66.6 to 82.39 mm SL. This correlation with SL has also been reported in other myctophid species^23,28,30,40,61^. However, although *N. resplendens* shows a standard length range greater than that in other myctophids, it has similar egg production, and consequently may have a higher energy cost for reproduction^39^. Although this study provides information on the reproductive biology of *N. resplendens*, we suggest that future research should study the fecundity and spawning season to understand the reproductive strategy of this species.

In the Canary Islands region, there is high fishing effort (artisanal and professional); however, although the fishes of the mesopelagic zones have no commercial interest, it is important to improve knowledge about this key fish group because they are prey for pelagic fishes (such as tuna) targeted by the fishing industry all along the West African coast. Because the myctophid species (such as *N. resplendens*) have a great abundance and biomass and because they are the link between secondary producers and upper trophic levels in the open ocean through the organismal migration^78^, they contribute significantly to the oceanic biological pump. In conclusion, the biological information provided for *N. resplendens* in this study (i.e., length frequency distribution, sex ratio, size at first maturity, spawning season and batch fecundity) together with information previously published about their age and growth^46^, contribute to expand the knowledge and the baseline for effective future management of this group of fish species.

## Data Availability

The datasets generated during the current study are not publicly available due to the data will be used by a student for a new degree research but are available from the corresponding author on reasonable request. Partial data was published in ‘‘Catálogo de especies meso y batipelágicas. Peces, moluscos y crustáceos. Colectadas con arrastre en las Islas Canarias durante las campañas realizadas a bordo del B/E ‘‘La Bocaina’’.

## References

[1] Benoit-Bird KJ, Au WWL (2002). Energy: Converting from acoustic to biological resource units. J. Acoust. Soc. Am..

[2] Simard Y, Mackas DL (1989). Mesoscale aggregations of euphausiid sound scattering layers on the continental shelf of Vancouver Island. Can. J. Fish. Aquat. Sci..

[3] Ariza A, Garijo JC, Landeira JM, Bordes F, Hernández-León S (2015). Migrant biomass and respiratory carbon flux by zooplankton and micronekton in the subtropical northeast Atlantic Ocean (Canary Islands). Prog. Oceanogr..

[4] Ariza A, Landeira JM, Escánez A, Wienerroither R, Aguilar de Soto N, Røstad A, Kaartvedt S, Hernándenz-León S (2016). Vertical distribution, composition and migratory patterns of acoustic scattering layers in the Canary Islands. J. Mar. Syst..

[5] Hays GC (2003). A review of the adaptive significance and ecosystem consequences of zooplankton diel vertical migrations. Hydrobiologia.

[6] Yatsu A, Sassa C, Moku M, Kinoshita T (2005). Night-time vertical distribution and abundance of small epipelagic and mesopelagic fishes in the upper 100 m layer of the Kuroshio–Oyashio Transition Zone in Spring. Fish. Sci..

[7] Olson, R. J. *et al. Bioenergetics, Trophic Ecology, and Niche Separation of Tunas*. (Advances in Marine Biology, 2016). 10.1016/bs.amb.2016.06.002 27573052

[8] Hudson JM, Steinberg DK, Sutton TT, Graves JE, Latour RJ (2014). Myctophid feeding ecology and carbon transport along the northern Mid-Atlantic Ridge. Deep Res. Part I Oceanogr. Res. Pap..

[9] Guidi L (2015). A new look at ocean carbon remineralization for estimating deepwater sequestration. Global Biogeochem. Cy..

[10] van Noord JE (2013). Diet of five species of the family Myctophidae caught off the Mariana Islands. Ichthyol. Res..

[11] Lam V, Pauly D (2005). Mapping the global biomass of mesopelagic fishes. Sea Around Us Proj. Newsl..

[12] Irigoien X (2014). Large mesopelagic fishes biomass and trophic efficiency in the open ocean. Nat. Commun..

[13] Hulley PA (1981). Results of the research cruises of FRV" Walther Herwig" to South America. LVIII. Family Myctophidae (Osteichthyes, Myctophiformes). Arch. für Fischereiwiss..

[14] Hulley, P. A. Myctophidae. In *Check-List of the FISHES of the Eastern Tropical Atlantic (CLOFETA)* (eds Quero, J. C., Hureau, J. C., Karrer, C., Post, A. & Saldanha, L.) 398–467 (1990).

[15] Lubimova, T. G., Shust, K. V. & Popkov, V. V. Specific features in the ecology of Southern Ocean mesopelagic fish of the family Myctophidae. In *Biological Resources of the Arctic and Antarctic (Collected Papers)* 337 (1987).

[16] Catul V, Gauns M, Karuppasamy PK (2011). A review on mesopelagic fishes belonging to family Myctophidae. Rev. Fish Biol. Fish..

[17] Cherel Y, Fontaine C, Richard P, Labat JP (2010). Isotopic niches and trophic levels of myctophid fishes and their predators in the Southern Ocean. Limnol. Oceanogr..

[18] Pérez-Rodríguez A (2012). An Integrative Study to the Functioning of the Flemish Cap Demersal Community.

[19] Battaglia P (2013). Feeding habits of the Atlantic bluefin tuna, *Thunnus thynnus* (L. 1758), in the central Mediterranean Sea (Strait of Messina). Helgol. Mar. Res..

[20] Rosas-Luis R, Villanueva R, Sánchez P (2014). Trophic habits of the Ommastrephid squid Illex coindetii and Todarodes sagittatus in the northwestern Mediterranean Sea. Fish. Res..

[21] Hedd A, Montevecchi WA, Davoren GK, Fifield DA (2009). Diets and distributions of Leach’s storm-petrel (*Oceanodroma leucorhoa*) before and after an ecosystem shift in the Northwest Atlantic. Can. J. Zool..

[22] Ohizumi H, Kuramochi T, Kubodera T, Yoshioka M, Miyazaki N (2003). Feeding habits of Dall’s porpoises (*Phocoenoides dalli*) in the subarctic North Pacific and the Bering Sea basin and the impact of predation on mesopelagic micronekton. Deep Sea Res. Part I Oceanogr. Res. Pap..

[23] Lisovenko LA, Prut’ko VG (1987). Reproductive biology of *Diaphus suborbitalis* (Myctophidae) in the equatorial part of the Indian Ocean. 2. Fecundity and reproductive potential. J. Ichthyol..

[24] Shotton, R. *Lanternfishes: A potential fishery in the Northern Arabian Sea. Review of the state of world fishery resources: Marine fisheries. FAO Fisheries Circular, (920).* (1997).

[25] Olivar MP (2012). Vertical distribution, diversity and assemblages of mesopelagic fishes in the western Mediterranean. Deep Res. Part I Oceanogr. Res. Pap..

[26] Battaglia P (2014). Diet of the spothead lanternfish *Diaphus metopoclampus* (Cocco, 1829) (Pisces: Myctophidae) in the central Mediterranean Sea. Ital. J. Zool..

[27] Lisovenko LA, Prutko VG (1986). Reproductive biology of *Diaphus suborbitalis* (Myctophidae) in the equatorial part of the Indian Ocean. 1. Nature of oogenesis and type of spawning. J. Ichthyol..

[28] Dalpadado P (1988). Reproductive biology of the lanternfish *Benthosema pterotum* from the Indian Ocean. Mar. Biol..

[29] Hussain SM (1992). The reproductive biology of the lantern fish *Benthosema fibulatum* from the northern Arabian Sea. Fish. Res..

[30] Gartner JV (1993). Patterns of reproduction in the dominant lanternfish species (Pisces: Myctophidae) of the eastern Gulf of Mexico, with a review of reproduction among tropical-subtropical Myctophidae. Bull. Mar. Sci..

[31] Gjosaeter J (1981). Life history and ecology of the myctophid fish *Notoscopelus elongatus Kroeyeri* from the northeast Atlantic. Ser. Havundersokelser Fisk. Skr..

[32] Kawaguchi K, Mauchline J, Mauchline J (1982). Biology of myctophid fishes (Family Myctophidae) in the rockall trough, Northeastern Atlantic Ocean Biology of Myctophid Fishes (Family Myctophidae) in the Rockall Trough, Northeastern Atlantic Ocean. Biol. Oceanogr..

[33] Hussain SM, Ali-Khan J (1987). Fecundity of *Benthosema fibulatum* and *Benhosema pterotum* from the northern Arabian sea. Deep Sea Res. Part A Oceanogr. Res. Pap..

[34] Young JW, Blaber SJM, Rose R (1987). Reproductive biology of three species of midwater fishes associated with the continental slope of eastern Tasmania, Australia. Mar. Biol..

[35] Prosch RM (1991). Reproductive biology and spawning of the myctophid *Lampanyctodes hectoris* and the sternoptychid *Maurolicus muelleri* in the Southern Benguela ecosystem. S. Afr. J. Mar. Sci..

[36] Clarke TA (1984). Fecundity and other aspects of reproductive effort in mesopelagic fishes from the North Central and Equatorial Pacific. Biol. Oceanogr..

[37] Flynn AJ, Paxton JR (2012). Spawning aggregation of the lanternfish *Diaphus danae* (family Myctophidae) in the north-western Coral Sea and associations with tuna aggregations. Mar. Freshw. Res..

[38] García-Seoane E, Bernal A, Saborido-Rey F (2014). Reproductive ecology of the glacier lanternfish *Benthosema glaciale*. Hydrobiologia.

[39] Sassa C, Ohshimo S, Tanaka H, Tsukamoto Y (2014). Reproductive biology of *Benthosema pterotum* (Teleostei: Myctophidae) in the shelf region of the East China Sea. J. Mar. Biol. Assoc. UK.

[40] Sassa C, Tanaka H, Ohshimo S (2016). Comparative reproductive biology of three dominant myctophids of the genus Diaphus on the slope region of the East China Sea. Deep. Res. Part I Oceanogr. Res. Pap..

[41] Eschmeyer, W. N., Fricke, R. & R, van der L. Catalog of Fishes, electronic version (3 January 2017). San Francisco, CA (California Academy of Sciences). (2018).

[42] Collins MA (2012). Latitudinal and bathymetric patterns in the distribution and abundance of mesopelagic fish in the Scotia Sea. Deep. Res. Part II Top. Stud. Oceanogr..

[43] Albikovskaya LK (1988). Some aspects of the biology and distribution of glacier lanternfish (*Benthosema glaciale*) over the slopes of Flemish Cap and eastern Grand Bank. NAFO Sci. Counc. Stud..

[44] Nafpaktitis, B. G. *Review of the lanternfish genus Notoscopelus (family myctophidae) in the north Atlantic and the Mediterranean*. *Bulletin of Marine Science* vol. 25 (1975).

[45] Hulley, P. A. & Paxton, J. R. Myctophiformes—Myctophidae/Neoscopelidae. In *FAO Species Identification Guide for Fisheries Purposes: The Living Marine Resources of the Eastern Central Atlantic* (eds Carpenter, K.E. & De Angelis, N.) 1922–1923, Rome (2016).

[46] Sarmiento-Lezcano AN, Triay-Portella R, Castro JJ, Rubio-Rodríguez U, Pajuelo JG (2018). Age-based life-history parameters of the mesopelagic fish *Notoscopelus resplendens* (Richardson, 1845) in the Central Eastern Atlantic. Fish. Res..

[47] Sutton TT (2017). A global biogeographic classification of the mesopelagic zone. Deep Sea Res. Part I Oceanogr. Res. Pap..

[48] Moser, H. G. & Ahlstrom, E. H. Myctophidae: lanternfishes. In *The Early Stages of Fishes in the California Current Region. Cal. Coop. Ocean. Fish. (CalCOFI).* (ed. Moser, H. G.) 387–475 (1996).

[49] Bordes, F. *et al.* Catálogo de especies meso y batipelágicas. Peces, moluscos y crustáceos. Colectadas con arrastre en las Islas Canarias, durante las campañas realizadas a bordo de B/E ‘La Bocaina’. Instituto Canarios de Ciencias Marinas (ICCM), Agencia Canaria de Investig. in 326 (2009).

[50] Bordes Caballero, F. *et al.* Epi- and mesopelagic fishes, acoustic data, and SST images collected off Lanzarote, Fuerteventura and Gran Canaria, Canary Islands, during cruise ‘La Bocaina 04–97’. In *Informes Técnicos del Instituto Canario de Ciencias Marinas.* 1–45 (1999).

[51] Wienerroither, R. M. *Species composition of mesopelagic fishes in the area of the Canary Islands, Eastern Central Atlantic. Informes Técnicos del Instituto Canario de Ciencias Marinas*. (2003).

[52] Fulton TW (1911). The Sovereignty of the Sea: An Historical Account of the Claims of England to the Dominion of the British Seas and of the Evolution of the Territorial Waters, with Special Reference to the Rights of Fishing and the Naval Salute.

[53] Brown-Peterson NJ, Wyanski DM, Saborido-Rey F, Macewicz BJ, Lowerre-Barbieri SK (2011). A standardized terminology for describing reproductive development in fishes. Mar. Coast. Fish..

[54] Brown-Peterson NJ, Overstreet RM, Lotz JM, Franks JS, Burns KM (2001). Reproductive biology of cobia, *Rachycentron canadum*, from coastal waters of the southern United States. Fish. Bull..

[55] Luna LG (1968). Manual of Histologic Staining Methods of the Armed Forces Institute of Pathology.

[56] De Vlaming, V. *Oocyte Developmental Pattern and Hormonal Involvement Among Teleosts*. (1983).

[57] Murua H (2003). Procedures to estimate fecundity of wild collected marine fish in relation to fish reproductive strategy. J. Northwest Atl. Fish. Sci..

[58] Kiesbu OS (1989). Oogenesis in cod, *Gadus morhua* L., studied by light and electron microscopy. J. Fish Biol..

[59] Hunter JR, Lo NCH, Leong JH (1985). Batch Fecundity in multiple spawning fishes. NOAA Tech. Rep. NMFS.

[60] R Core Team. R: A Language and Environment for Statistical Computing. (2018).

[61] Sassa C, Hirota Y (2013). Seasonal occurrence of mesopelagic fish larvae on the onshore side of the Kuroshio off southern Japan. Deep. Res. Part I.

[62] QGIS Development Team. QGIS Geographic Information System. Open Source Geospatial Foundation. (2020). https://www.qgis.org/es/site/.

[63] Wienerroither R, Uibleina F, Bordes F, Moreno T (2009). Composition, distribution, and diversity of pelagic fishes around the Canary Islands, Eastern Central Atlantic. Mar. Biol. Res..

[64] Olivar MP (2018). Variation in the diel vertical distributions of larvae and transforming stages of oceanic fishes across the tropical and equatorial Atlantic. Prog. Oceanogr..

[65] Clarke TA (1983). Sex ratios and sexual differences in size among mesopelagic fishes from the central Pacific Ocean. Mar. Biol..

[66] Herring PJ (2002). The Biology of the Deep Ocean.

[67] Dalpadado P (1983). Aspects of the Biology of *Benthosema pterotum* (Myctophidae) from the Indian Ocean.

[68] Filin AA (1997). Growth, size and age composition of the *Notoscopelus kroeyerii* (Myctophidae). J. Ichthyol..

[69] Greely TM, Gartner JVJ, Torres JJ (1999). Age and growth of *Electrona antarctica* (Pisces: Myctophidae), the dominant mesopelagic fish in the Southern Ocean. Mar. Biol..

[70] Hulley, P. A. Myctophidae. In *Fishes of the North-eastern Atlantic and the Mediterranean* (eds Whitehead, P.J.P. *et al.*) 429–483 (1986).

[71] Kawaguchi K, Shimizu H (1978). Taxonomy and distribution of the lanternfishes, genus Diaphus (Pisces, Myctophidae) in the western Pacific, eastern Indian Oceans and the southeast Asian Seas. Bull. Ocean Res. Inst. Univ. Tokyo.

[72] Herring PJ (2007). Sex with the lights on? A review of bioluminescent sexual dimorphism in the sea. J. Mar. Biol. Assoc. UK.

[73] Barnes AT, Case JF (1974). The luminescence of lanternfish (Myctophidae): Spontaneous activity and responses to mechanical, electrical, and chemical stimulation. J. Exp. Mar. Biol. Ecol..

[74] Clarke TA (1973). Some aspects of the ecology of lanternfishes (Myctophidae) in the Pacific Ocean near Hawaii. Fish. Bull..

[75] Karnella C (1983). The Ecology of Lanterfishes (Myctophidae) in the Bermuda ‘Ocean Acre’.

[76] Sabatés A, Masò M (1990). Effect of a shelf slope front on the spatial distribution of mesopelagic fish larvae in the western Mediterranean. Deep. Res..

[77] Alekseyeva YI, Alekseyev FY (1983). Some aspects of reproductive biology of the lanternfishes, Myctophum punctatum and *Notoscopelus resplendens* (Myctophidae), from the eastern tropical Atlantic. J. Ichthyol..

[78] Davison PC, Checkley DM, Koslow JA, Barlow J (2013). Carbon export mediated by mesopelagic fishes in the northeast Pacific Ocean. Prog. Oceanogr..

